# Hæmorrhoids and Their Treatment

**Published:** 1881-05

**Authors:** A. Charge

**Affiliations:** Paris


### HÆMORRHOIDS AND THEIR TREATMENT.

BY DR. A. CHARGE, PARIS.

(Continued from page 145.)

### *Treatment: Special Indications.*

Aconite Nap.—It is most generally taught that *Aconite* has no place in the treatment of hæmorrhoids; a study of the pathogenesis of this remedy and its clinical record teaches us the contrary. Under it we find: itching in rectum and anus; tenesmus of rectum; pain in rectum; stinging and constant pressure in rectum and at anus. In these symptoms, we have evidence for the use of *Aconite* in hæmorrhoids.

*Aconite* is the more indicated if the patient be young, plethoric, especially when the hæmorrhoids are inflammed, bleeding, very painful and protruding, accompanied by fever; or when the local inflammation produces a discharge of whitish mucus, accompanied by heat and incessant itching at anus and surrounding parts. *Aconite* is also indicated by the following group of symptoms: cephalalgia pressing from within outward, pressure in the temples with vertigo and weakness of memory; nausea and sleepiness after eating; scanty urinary excretion accompanied by violent tenesmus. Small appetite and thirsty; pressure in region of the liver; pains in lumbar region; troubled sleep.

Aesculus Hipp.—Painful hæmorrhoids, which are prolapsed on account of the constipation, with but very little bleeding; sensation of fullness in rectum which causes frequent desire to go to stool; considerable swelling, and dark purple color of hæmorrhoidal tumors, dryness of rectum, with burning, smarting pain in the loins and hips with pressure below.

The history of the horse-chestnut is so curious that we may be pardoned for inserting it here, parenthetically. The attentive reader will gather three facts from these new proofs.

It amounts to nothing for the therapeutist to turn indefinitely our attention to the analysis of the constituent parts of medicines. Example: skillful analysis of *Aesculus hipp.* shows that the bark of the young branches of the horse-chestnut contains some tannin, some fraxine or paviine which are changed by acids into fraxetine, glucose and another glucoside. Conzonieri found a non azotic substance, yet alkaline, and able to combine with *Sulphuric acid*, forming a chrystallized salt like silky needles. Wurtz places it among the glucosides; its aqueous solution is blue by reflection, colorless by transmitted light. Aesculine changes into æsculitine and glucose; it is a little bitter, and slightly deliquescent in the air. (Cauvet, Hist. Nat. Med.) These observations may be very interesting to the chemist, but I am positive of their uselessness to the therapeutist. They have beautifully described these points in works bearing such a title as "The History and Properties of Plants useful to Man." No one can convince me that the useful properties of plants are contained solely in their color, etc. This materialism is done away with; and a science capable of teaching us the curative action of medicines has now all to do; unless we take as serious the stammerings of ignorance and intolerance in a field where facts alone ought to speak. "The bark of the young branches of the horse-chestnut has been recommended as a febrifuge, its decoction has sometimes served as an antiseptic in wounds of a bad nature. They call it (*Aesculine*) a febrifuge and also prescribe it for periodical neuralgias." (*Cauvet, eod. loc.*). What therapeutics! based on *ipse dixit*, chance and empiricism! It is not necessary for us to credit such dubious teaching as we find in our classic works, now that the logic, good sense and genius of Hahnemann have shed a great light upon therapeutics.

"There is no method more safe or more natural for obtaining reliable knowledge of the proper effects of medicines, than to administer each drug separately and in small doses to healthy persons and to note the symptoms occurring, physical and mental; that is, the symptoms of disease each medicine is able to produce." (Hahnemann's "Organon," § 108) A different method has taught the use of *Aesculus hipp.* The regular school is ungrateful to empiricism, which has given it much, and many of the methods borrowed from empiricism have become the most beautiful flowers in its crown. But, in spite of all that, while keeping all it has gathered from chance the regular school, proud and conceited, has none the less sworn an implacable hatred against empiricism. For a long while *Aesculus hipp.* has been used as a domestic remedy for hæmorrhoids. "For a long time the inhabitants of India (East) have considered the fruit of the *Aesculus hipp.*, as a preventative against hæmorrhoids. This prophylactic action is due to the miasmatic emanations of this fruit." (Dr. Dibot, *hæmorrhoids and their treatment*, Paris, 1881).

Besides, it is not only the prophylactic action which is so constant; its curative power is wonderfully and surely demonstrated. "A member of the Institute, brother of one of our most celebrated dramatic authors, and himself a distinguished writer, of a very healthy complexion and sanguineo-bilious temperament, born of a hæmorrhoidal parent, had suffered several times in his youth with irregular attacks of hæmorrhoids. When about twenty-eight or thirty years old, he was attacked by hæmorrhoids which lasted two years, with fearful pain and such a continued and abundant discharge that he wondered at his strength lasting under it. While this trouble was at its worst, one day walking through a grove of horse-chestnut trees, his hostess advised him to place some of the nuts in his pocket, saying it would cure him. After some joking about such a singular remedy, he placed five nuts in his pocket, simply to please her. The next day, without thinking of the horse-chestnuts, he was most agreeably surprised not to experience, on going to the water-closet, a renewal of the pains he was accustomed to suffer, for the pains were much less. We can easily imagine that he then remembered the horse-chestnuts and was careful not to throw them away! The improvement continued, and so rapidly that at the end of three or four days he was completely cured. Since then, that is, for twenty-six or twenty-seven years (he is to-day fifty-seven), he has not experienced the least trouble and generally enjoys the best health, this in spite of sedentary work, very troublesome and surely a great cause of these sufferings." (*Montegre, Dict. des Sciences Med.*, vol. xx, p. 437).

It had been my intention to curtail this case and not relate it in full as given by Montegre. But there is all through it such an air of honesty and conscientious observation, that one can not doubt it. He himself vouches for its truth. So we are much more advanced than the inhabitants of East India; from the prophylactic power of the horse-chestnut we have derived a complete demonstration of its curative action, as shown in a case, most remarkable for its intensity and chronicity. . . . .

*Aesculus hipp.* has been thoroughly experimented with by man and with this result: congestion of rectum, hæmorrhoidal tumors as large as a nut, of a dark purple color and very painful, with a burning sensation. Desire to strain at stool for a long time; pains in sacrum and buttocks; constant desire to go to stool, with fullness and pressure in rectum; difficult evacuation of hard fæces caused by dryness and constriction of rectum; violent pains in rectum, abdomen; lumbar region, etc.* How, after these symptoms can one refuse to admit the great similarity of the symptoms of *Aesculus hipp.* to those of hæmor-

* *Aesculus* has some very characteristic symptoms: "Dry uncomfortable feeling in the rectum, which feels as if filled with little sticks; excessive dryness with sensation of heat;" mucous membrane of rectum seems to be thickened or stiffened and so to retard passage of stool; has soreness, burning, itching, etc., hæmorrhoids are like ground nuts of purple color; stools generally hard with pain in back, also a liquid stool, white as milk; the fullness and itching at anus are worse when walking, also backache.—*Trans.*

rhoids, in a certain number of cases. It is not upon the custom of the inhabitants of India, nor even from the cure of the member of the Institute which we heard to-day, it is on the pathogenesis of the drug, our corner-stone, that we build its curative powers. Carrying horse-chestnuts in one's pocket has prevented, and even cured, certain cases of hæmorrhoidal disease of long standing; this double action—prophylactic and curative—some may attempt to explain as due to miasmatic emanations from the horse-chestnut.

As that explanation serves as well as any other, I will not contest it. I will only say with Montegre: "There is a great difference between a fact and the hypothesis by which it is attempted to explain it; facts will always be admitted when confirmed by sufficient evidence, the explanations will be made when they are able to do so." (*Dict. des Sc. Med.*, p. 632).

One last word; when our learned men are willing to reflect deeply on the power of the miasmatic emanations of the horse-chestnut, we shall find them less opposed to our dilutions and globules. These are, at least, tangible, and the emanations are not. Among the physicians who espouse the same cause as we do, perhaps these miasmatic emanations, considered in the double character of their reality and their efficacy, may cause them to adopt a posology more Hahnemannian.

Aloes.—Local symptoms; hæmorrhoids prolapsed, painful, with heat and tenesmus; tumors very large, constant pressure in rectum. Pain, pulsation, itching and heat at anus. Hæmorrhoids protrude like grapes, they burn and bleed during and after stool. Cutting pain at anus and through rectum into abdomen. Ameliorated by cold water. Profuse emission of gas before each stool. The rectum constantly secretes mucus which escapes from anus, even with attempts at defecation. While *Sulphur*, *Aesculus* and *Collins.* have constipation as an indication, in hæmorroids, *Aloes*, is especially indicated in diarrhœa.

General Symptoms.—A headache characterized by a dull pain, pressing in the frontal region; not very annoying except for the difficulty of thinking which follows. A learned surgeon of Paris has recently declared that *Aloes* has no special (congestive) action upon the hæmorrhoidal veins; we are surprised at this for experience contradicts it, and also we give credit to the following testimony of Trousseau: "Two grains are sufficient to promptly cause a slight irritation of the rectum, which will certainly produce a hæmorrhoidal flow. We are able in a certain number of cases, to cause an active irritation in the lower part of the intestine and a heaviness in lower part of abdomen; sometimes even a discharge of blood sufficient to have come only from the hæmorrhoidal veins. The use of this drug causes various serious affections." (p. 763).

*Trousseau* (*eod. loc*)., lays the peculiar blame on *Aloes* that it can not cure real hæmorrhoidal tumors. It is possible. How does the vital power affect the organism so as to cause hæmorrhoids? We do not know and probably will always be ignorant of it. But we do know very well that hæmorrhoids are caused by some particular predisposition. This predisposition is not so well combatted by *Aloes*, but is most effectually combatted by *Sulphur*. Hence *Sulphur* should not be overlooked in the treatment of hæmorrhoids.

Alumen, (Bi-sulphate of aluminum and potassium).—Some physicians of our school praise *Alum* as the equal of Hamamelis in hæmorrhoidal hæmorrhage, recollecting, perhaps, the astringent power inherent in *Alum*, which has been lauded beyond measure by the old school and used for a long time in washes and suppositories. For us, who only use the primary effects of a drug as symptoms which the medicine will cure; for us, who have learned from Hahnemann that medicines will cure diseases whose symptoms are similar to those of the remedy; for us who are convinced that in all cases and for all remedies "that *that* drug proved in its effect upon healthy persons, to produce the greatest number of symptoms similar to those found in the case of disease to be treated, and when administered in properly potentized and diminished doses, will rapidly, thoroughly, and permanantly cancel and turn into health the totality of the symptoms of the present case of disease." (The "Organon," § 25). To us, the astringent property of *Alumen* is valueless, and the pathogenesis of the drug does not authorize us to utilize its hæmostatic properties.

The use of *Alumen* is confined to the following symptoms: tumors prolapsed after hard stool; sharp pain inside rectum; violent itching around anus, evening and night especially; severe pains in anus during stool and for some minutes after, ameliorated by bending forward and by pressure; (worse lying on side), ulcers of rectum, with ichorous, fœtid or sanguinolent discharge. *Dyspnœa during efforts at stool;* asthmatic troubles with hæmorrhoids. Weakness of neck of bladder with incontinence of urine. There is reported, in "Allen's Encyclopædia," the following case: "Frequent attacks first hard, then soft stools, and after it for three hours, the most violent pains, burning, shooting, and particularly cutting in the rectum upward, lasting from morning till noon; with the stool passes a great deal of blood, without any relief; sometimes a sensation as if the anus would protrude. After the patient had carried a piece of Alum in his breeches pocket, he never had it again. (Reported to Dr. Neidhard). Same symptoms have in many cases been cured by giving high potencies. (200th, Jenichen)."

ALUMINA (Aluminum oxide.)—Complete inertia of rectum; *seems paralyzed* and inactive as from deficient peristaltic power; soft stool hard to evacuate, must strain; stools hard and dry as from want of proper intestinal secretions; after stool dropping of blood, or the blood spurts out. Pain in rectum, lasts long; itching, pricking and burning at anus; hæmorrhoids prolapsed by standing or walking, worse in evening, ameliorated by the night's rest. Crawling sensation at anus as from worms. Sweat on perineum with intolerable itching which is increased and becomes painful from scratching.

AMM. CARB.—Tumors painful to touch but not inflammed; bleeding at each stool; the discharge consists of pure blood, without any muco-purulent matter. Constipation. Indicated in case of children, old men and in pale subjects, with bloated face, weak and inclined to be sad, troubled by constant thought of death, which causes continual misery. These concomitant symptoms are of characteristic importance; pustules on nose and face.

AMM. MUR.—Constipation is less marked than under *Amm. carb.*, when present stools are hard and crumbling, require effort to pass them (Hg.); hæmorrhoids protrude during stool, or in the intervals between efforts at stool, are sore and smart. During and after stool, even when fæces are soft, there is heat in rectum; pustules around anus itching and painful; sticking and cutting pains in perineum, while walking and in the evening. Weakness, debility; frequent sensation of weakness or faintness in morning and after dinner. Hæmorrhoids from suppressed leucorrhœa (Hg.).

